# On the Causes of Epidemical Diseases

**Published:** 1832-03

**Authors:** Francis Adams

**Affiliations:** Surgeon, Banchory Ternan, Aberdeen.


### EPIDEMICAL DISEASES.

### *On the Causes of Epidemical Diseases.* By Francis Adams, Esq., Surgeon, Banchory Ternan, Aberdeen.

An epidemical disease is a disease which seizes upon a number of persons at a time, or during a particular period: an endemical disease is a disease to which the inhabitants of any district or country are subject: a sporadic disease is one which is confined to individuals. In these senses the terms are used by the best authorities, both ancient and modern, and it is therefore to be regretted that some popular writers, of late, should have ventured to alter their original import. Epidemical diseases partake, for the most part, of the febrile character; such as smallpox, measles, scarlet fever, the plague, influenza, dysentery, and the like. Endemical diseases are commonly of a chronic nature; as bronchocele, which prevails in the Alpine districts, the plica Polonica in Poland, and scrofula in Spain; and yet acute diseases are endemical in some places, as the malaria in certain parts of Italy. Most diseases of a purely inflammatory nature are sporadic; as pleurisy, phrenitis, enteritis, and hepatitis. I mean to confine my attention at present to the consideration of the first class, namely, the epidemics; and shall therefore proceed to give a condensed view of the various causes which have been assigned for their origin. In doing this, I shall adopt the arrangement of the causes of pestilential diseases laid down by FABIUS PAULINAS, in his *Prælectiones Marciæ;* since, although somewhat arbitrary, it has the merit of being the most methodical and comprehensive arrangement which I have met with. Paulinus, then, has arranged the causes of these disorders under the heads of the Divine, the Elementary, and the Material: the precise signification of these terms will be explained as we go on.

1st. *Of the Divine.* A general propensity has prevailed, in all ages and in all countries, to account for popular distempers, upon the supposition that they are the ministers of divine wrath, commissioned to punish the wickedness of mankind. In the Iliad of HOMER, the pestilence which attacked the Grecian camp before Troy is ascribed to the vengeance of the offended god of day. In allusion to it, CELSUS says of the poet, "eodem auctore disci potest morbos tum ad iram deorum immortalium relatos esse, et ab iisdem opem posci solitam." Many of the ancient historians, also, (such as LIVY, DIODORUS SICULUS, DIONYSIUS the Halicarnassian, AGATHIAS, and PROCOPIUS,) agree in occasionally referring the epidemics which they mention, to the wrath of the gods, although they sometimes attribute them to natural causes. It appears likewise, from THUCYDIDES, that the same superstitious belief prevailed in his time. In like manner many modern authors, as, for example, GEORGIUS AGRICOLA, CARDANUS, and BAPTISTA CODRONCHIUS, refer certain pestilential epidemics to the operation of demons; and RAIMUNDUS DE VINARIO, ARNOLDUS DE VILLA NOVA, and many writers of that age and of succeeding ages, referred them to the malign conjunction of the planets; that is to say, to a supernatural cause. Even the Scripture appears to give some countenance to this supposition: thus it is said that the pestilence which proved so fatal to the Jews in the days of King David, was a punishment inflicted by heaven, because he had taken a census of the tribes.* This last authority would, no doubt, be held quite decisive of the question,

* 2 Samuel, chap. xxiv. The account of it given by JOSEPHUS contains a mixture of mythological and natural images, similar to that in Homer's description, and such as we often meet with in the ancient poets. (Hist. vii. 13.) It is very properly remarked by the learned THOMAS BURNET, (Epistola de Archæologiis Philosophicis,) that there are many passages in the Old Testament, which, although they appear at first sight to be purely narrative, can only be understood in a figurative sense, agreeably to the ancient mode of Prosopopœia. In this manner we must surely explain the 1st and 3d chapters of Hosea, and 1 Kings, xxii. 19—22. He says afterwards, "Nec id cuiquam proinde vertendum esse vitio quod a verbis recedat, ubi literalis interpretatio Cæsura esset Magestatem divinam, aut rerum naturæ palam obnititur."

provided the words of the historian are to be strictly taken in their literal sense; but we must not forget that the writers of the East indulge very much in the allegorical mode of narration, and that the Jews, in particular, had a propensity to explain natural causes by referring them to the immediate agency of supernatural beings. Thus, thunder is called by Job "the voice of God;" the rainbow is said to be "the bow of God;" and in the Psalms a term is applied to the plague, which signifies "the arrow of the angel of death;" and thus also, agreeably to the popular belief, the strange convulsions and mental emotions of epileptics and maniacs were supposed to be produced by the agency of demons."* For my own part, I am convinced that HIPPOCRATES has correctly said, that "no disease comes from the gods, one more than another, each acknowledging its own natural and manifest cause." With this high authority, LUCRETIUS, GALEN, and most of the philosophers of antiquity, agree; maintaining that all diseases originate in natural causes, and entirely rejecting the popular superstition which looked upon them as marks of the divine displeasure: indeed, I have often thought that the expression "anger of God" ought not to be taken, as is too frequently done, in a literal sense; for if, as philosophy† and religion teach us, the Divinity be a Being unchangeable and wholly devoid of passions, it is not easy to define the import of these words as applied to Him, or to attach any precise meaning to them. At all events, it ill becomes a frail mortal to attempt to penetrate the councils of the Almighty, or to determine which of the casualties of life are tokens of his favor, and which of his high displeasure; and is there not some inconsistency in maintaining that the Creator acts in violation of the precept which he enjoined on his creatures, "not to do evil, even that good may arise."

It was well said by an ancient sage, that, as a mark is not set up to be missed, so there is nothing naturally evil in the world;‡ and Dr. PALEY, in like manner, says that there is no contrivance in the universe, the object of which is evil. If, then, God has formed no apparatus in the tooth for the pur-

* V. LINDINGER, de Dæmoniacis, and MEAD, Sacra Medica. It appears from the Hippocratic treatise, de Morbo Sacro, that the common people in Greece, as well as in Judea, believed that epileptic convulsions are produced by the agency of demons.

† V. PLATO, Epinomis; ARISTOTELIS, Metaphys. lib. xii.

‡ EPICTETUS, Enchiridion. The commentary of SIMPLICIUS on this passage contains the most sensible disquisition I have ever met with on the origin of evil, both natural and moral. MAXIMUS TYRIUS adopts nearly the same views of the subject in one of his Declamations.

pose of making it ache, so we may be assured that he has created no morbific particles for the purpose of engendering pestilential disorders.

Enough, I trust, has been said to justify us in unreservedly rejecting the supernatural origin of diseases, as a tenet inconsistent with genuine religion, and tending to encourage a debasing superstition. At the same time, we would not be understood to condemn all religious acts of devotion performed during the prevalence of a pestilential malady.* To live at all times under a solemn sense of an all-ruling Providence, is, no doubt, the duty of every creature that is made capable of in anywise comprehending the existence of a superior Power; and such religious services as, in the day of affliction, are calculated to impress mankind with a rational confidence in his beneficence, are far from being blameworthy, provided they do not foster a superstitious belief that the Ruler of the Universe distributes rewards and punishments under the influence of such passions as those which agitate the human breast, or divert us from the study of those natural causes to which such distempers ought undoubtedly to be traced. Though we find no fault with the man who betakes himself to supplication and prayer during a violent thunderstorm, yet no sensible person, on such an occasion, expects that his religious services will serve him instead of the metallic tractors, invented by modern science for turning aside the electric fluid; and so, in like manner, although the pious may be allowed to repose unbounded confidence in devotional performances, when threatened by the near ap-

* The learned and pious Diemerbroeck, represents the Prophylaxis Theologica as the first and most important consideration during a pestilence; and, with this intention, directs to deprecate the Divine vengeance by public and private rites of devotion, and by fasting and eleemosynary acts. This practice appears to have been followed in the most ancient times. Livy repeatedly makes mention of supplications to the gods, for one or more days, having been appointed by the Roman senate on account of the plague. In Catholic times, supplications to the Virgin Mary were usually practised on such occasions; and, if we may believe the Fathers of the church, they were often attended with the most signal efficacy: many such histories are collected from their writings by the indefatigable Fabius Paulinus, Marciæ Prælect. lib. ii. It is a singular example of the proneness of the human heart to run into extremes, that it has often been remarked that when the inhabitants of a city found their devotional acts of no avail in removing the pestilence, they relapsed into the most wanton profligacy and debauchery. Thucydides relates that this was decidedly the case in Athens during the memorable plague described by him; and a similar account is told of the dissipation of Genoa during a fatal pestilence in the seventeenth century. From the licentiousness of many of the tales in the Decameron of Boccacio, it is obvious that the morals of the inhabitants of Florence had become extremely profligate during that pestilential season.

proach of a fatal epidemic, the well-informed will seek to discover the natural causes of the disease, and endeavour to ward off its attack, or alleviate its violence, by such remedial means as are suggested by reason, experience, and analogy.

2d. *Of the Elementary.* Before entering upon the discussion of these, it will be proper to explain, as briefly as possible, the ideas entertained by the ancient philosophers regarding the elements.* Aristotle taught, or, I should rather say, *demonstrated,* that the primary matter from which all things are formed is a substance devoid of all quality and form, but susceptible of all forms and qualities. This "receptacle of forms," as it was called by Plato, is said, in the language of the peripatetic philosophy, to be every thing in capacity, and nothing in actuality. It is the grand passive principle, from which all things are fabricated by Mind, the grand active principle and efficient cause: the one is possessed of universal privation, and the other of universal energy; it is the one which impresses, and the other which receives the forms of all things, which, being all constructed from the same fundamental ingredient, were held to owe their characteristic qualities not to their matter, but their forms. The elements, then, are the specific forms under which the first matter is presented to the sense of touch. That form which constitutes solidity the philosophers called Earth, under which they classed all metals, stones, and the like; for all these they held to be allied in nature, and to be only different modifications of the same form. The next form under which matter is presented to the senses is that which constitutes fluidity, called Water, and comprehending not only the native element, but every similar modification of matter, such as the juices of vegetables and the fluids of animals. The third form is Air, under which was classed every thing that is possessed of aerial consistence, such as the atmospheric air and all vapours and gases. The last form is Fire, which is matter in its extreme state of tenuity, comprehending lightning and the solar light. Instead, therefore, of maintaining, as has been represented, that the elements are

* For a fuller account of the ancient opinions regarding the elements, I would refer to the following authors: Aristotelis, Auscultationes Naturales, lib. iv., Meteorologica, i. 3.; Plato, Timæus; Ocellus Lucanus, de Universo; Timæus Locrus, de Anima Mundi; Diogenes Laertius, in Vita Pythagoræ; Iamblichus, in Vita Pythagoræ; Cicero, de Natura Deorum, ii. 7.; Ovidius, Metamorph. lib. xv.; Plotinus, Ennead, ii. c.6; Apuleius, de Philosophia Naturali; Galenus, de Elementis, lib. i.; Plutarchus, de Placitis Philosoph.; Marcus Antoninus, lib. iv. § 46; Philo Judæus, de Mundi Creat.; Haly Abbas, Theoricæ, i. 5.

"distinct, uncompounded, and immutable principles," the ancient philosophers held, on the contrary, that they are "all but different magnitudes, figures, and arrangements of particles of the same kind,"* and that they are all transmutable into one another. This generic explication will serve to convey such a notion of the ancient opinions concerning the elements as will supply our present purpose. I need scarcely add, that the physical system of the ancients remains unshaken by all the late discoveries of chemical science.

First, then, of *Fire* as a cause of epidemics. The celestial bodies which supply us with light and heat, and further exert a manifest influence upon the tides, the winds, and the rains, have been supposed, by certain distinguished physiologists, to be concerned also in producing many of those natural changes and derangements which take place in the animal system. Thus GALEN, who is not in general disposed to have recourse to far-fetched causes to explain the phenomena of health and of disease, can account for the crises in fevers in no other way but by supposing them to be regulated by the changes of the moon. The truly learned and judicious Dr. MEAD, in an interesting treatise "on the Influence of the Sun and Moon upon Human Bodies," has collected many curious facts to prove the power of the heavenly bodies in affecting the health. To this work we refer for further information on this subject. DIEMERBROECK, as he mentions, observes, that the plague at Nimeguen, in the year 1636, always raged with the most fatal effects about the new and full of the moon.† For the same reason, eclipses, which happen only at the conjunction and opposition of the sun and moon, have been supposed to be connected with the origin of epidemics. THUCYDIDES relates that there was an eclipse of the sun, in the summer in which the plague of Athens broke out; and similar coincidences are mentioned by other historians entitled to all credit. I might draw many such statements from the works of the earlier modern writers on medicine; but as their authority (whether deservedly or not I shall not take upon me to determine,) is now held in little estimation, I should most probably waste my time and la-

* Allusion is here made to Dr. WATSON's erroneous statement of the peripatetic doctrines concerning the elements, in his essay on the Transmutability of Water into Earth. (Chemical Essays, vol. iv., Essay 7th.)

† De Peste, c. 5, and 15. JOUBERTUS and JOANNES MORELLUS confirm this statement. According to Diemerbroeck, however, the moon is not the efficient cause, but only the sign of certain changes which take place in the earth and in the animal frame. Lord MONBODDO entertained a similar notion: see Ancient Metaphysics, vol. i.

bour in attempting to persuade the reader to allow them an impartial consideration. For my own part, although I cannot say I am satisfied that any of the authors I have alluded to has made out a strong case to prove the truth of his opinions, there certainly appears to me to be no absurdity in the notion that the laws of disease may be materially affected by planetary influence, although its effects are such as do not readily admit of calculation. It is well remarked by the profound philosopher PLOTINUS,* that the nature of a country is much influenced by its exposure to the sun, and that the animal and vegetable productions thereof participate in this influence; nay, that the species, magnitudes, passions, diseases, dispositions, and pursuits of mankind are very much influenced by this cause. Now, admitting that the planetary bodies, by their light, heat, or other sensible properties, produce the changes we have mentioned, it appears to me by no means improbable that they may likewise be concerned in the origin of epidemics. At all events, we are sure that atmospheric distemperature has a great effect upon the origin and progress of popular diseases, and the changes which the atmosphere undergoes are unquestionably much regulated by the sun and moon.

And here, perhaps, I ought to notice the electrical state of the atmosphere, which some have supposed to be connected with the origin of pestilential distempers. Many of the older writers mention that lightning, meteors, and various atmospherical appearances, have been observed to precede the plague. These phenomena were ascribed by the ancient philosophers to unequal collections of the elemental fire in the clouds, and of late have been referred to the different states of their electricity. FRACASTORIUS remarks, that they may all be considered as indications that the atmosphere is loaded with putrid vapours. DIEMERBROECK relates that the plague of Nimeguen was preceded by falling stars and nocturnal lightnings.†

2d. Of *Air* as a cause of epidemics. The sensible qualities of the atmosphere, namely, its heat, coldness, humidity, and dryness, have long been supposed to operate extensively in occasioning epidemical distempers. In the case of the Grecian plague at Troy, although, after the manner of the ancient poets, HOMER ascribes it to the interference of a god, he is understood by his scholiast, by EUSTATHIUS, MACRO-

* Ennead, iii. lib. i.

† De Peste, c. vi. He quotes Livy, Mercurialis, Forestus, Trichemius, Andreas Gallus, Crusius, and many other authors, who had made similar observations.

BIUS, HERACLIDES PONTICUS, and AMMIANUS MARCELLINUS, to have meant to represent it as deriving its origin from the atmosphere being overheated by the sun. HIPPOCRATES, in describing the constitution of the season in which the Athenian plague prevailed, marks it as being hot and humid, and without winds.* GALEN attributes it to a hot intemperament of the atmosphere.† HIPPOCRATES, in one of his Aphorisms, enumerates continual, ardent, tertian, and quartan fevers, cholera, diarrhœa, ophthalmy, and certain other diseases, as being the epidemics of summer. In like manner, extreme drought or dryness of the air has been supposed to produce diseases, as in the case of the plague described by LIVY, which happened in the 363d year of the city.‡ The poets also frequently mention the drought of summer as the cause of pestilence. (See the Œdipus of SENECA, and VIRGIL, Æneid, iii.) LIVY describes a fatal epidemic, which he attributes to a cold and snowy winter; and HIPPOCRATES and AVICENNA ascribe certain popular diseases to the same cause. FABIUS PAULINUS quotes ANDERNACUS, ANDRÆAS GALLUS, GRATIOLUS, and others, to prove that the plague may be produced by extreme cold. It is probable that it was cold, joined to a deficiency of food, which occasioned the epidemical fever that attacked the remains of the French army, upon their return from Moscow. Humidity of the atmosphere, especially when combined with heat, by promoting putrefaction, has always been held to cooperate in engendering pestilential diseases: to this cause BLONDUS attributes a plague, which he describes. So, also, Dr. MEAD says, "Such an alteration of the common air as renders it in a manner mephitical, (which is done by too much heat, and at the same time too great a proportion of watery and other grosser particles mixed with it,) may be the cause of epidemical diseases."§ It is to be remarked, however, that these constitutional characters operate most decidedly in warm climates, such as Greece and Asia; for in this country, where the weather is so inconstant, it is difficult to connect the temperaments of the seasons with the prevailing epidemics: hence our SYDENHAM complained "that he had made no progress in discovering the causes of epidemical diseases by observing the manifest qualities of the air, as having remarked that in different years, which agreed perfectly well in the visible temperature of the air, the raging diseases were very different, and so on the contrary:" and Dr. MEAD

* Epidem. iii. § 3.

† De Diff. Febr. lib. i.

‡ Hist. lib. v.

§ On Poisons.

likewise holds that, in this country, "although the manifest qualities of the air have a considerable share in producing epidemic diseases, there are other conjunct causes which alter the force of those qualities, either by increasing or diminishing them." However, as Dr. ARBUTHNOT states,* there can be no doubt that, upon any supposition regarding the nature of contagion, the chief instrument in propagating it is air: "this must be true," he adds, "from what cause soever you deduce the plague: if from an animate cause, as from invisible insects, the favorable constitution of the air to their propagation must be supposed; if from infection of any kind, the air is the medium through which it is propagated, and must favor it more or less in different seasons, because, by the change of the qualities of the air, it is quite extinguished, and, generally speaking, by cold." Dr. RUSSEL held that the constitution of the air favors the propagation of a pestilential virus, (of the nature of which we will treat by and by,) not by heightening its malignity, but by increasing the disposition of the body to receive contagion.† MURATORI entertained a similar opinion.

That air becomes deleterious, so as to occasion febrile diseases from stagnation alone, is stated decidedly by AVENZOAR:‡ he remarks, that the air of certain grottoes proves suddenly destructive of life, by acting upon the heart. That a calm operates in producing a morbific state of the atmosphere, is remarked by HIPPOCRATES in his Epidemics, and by his commentator GALEN.§

The winds, on the other hand, have been supposed instrumental in spreading diseases, by wafting the impure air from one place to another. GALEN considers that the plague of Athens was carried from Egypt to Attica by the winds; and so also ARCULANUS affirms that, in the year 1466, the plague was wafted across the Adriatic from Illyrium to Venice. I may add, that the pestilential cholera of the present day has been supposed to have been wafted over tracts of land by the tradewinds.

This will be the proper place to notice the conjecture that plagues are occasioned by animalcules floating in the atmosphere. To this cause BRADLEY attributes the famous plague of Marseilles in 1720, and that of London in 1665: he rests his hypothesis principally upon the fact that fumigations with aromatic and resinous substances were found to be the best preservatives from the disease. This doctrine, however, found

† On the Plague of Aleppo.

‡ De Epidemicis.

§ Epidem. cum Comment. Galen, iii. § 3.

advocates in KERCHER and LINNÆUS. DONDI, as Dr. WINTERBOTTOM informs us, holds that contagion is matter endowed with a vital principle, and resembles parasitic seeds. Dr. MEAD remarks, that some refer the propagation of contagion to certain ova, or eggs; but he thinks that the supposition is grounded upon no manner of observation. In short, all these hypotheses are of such a nature that they cannot be readily proved nor disproved. I may remark, however, that the circumstance related by Diemerbroeck and others, that pestilential diseases sometimes rage with unabated fury during the cold of winter, is utterly irreconcilable with the supposition of an animal or vegetable origin.*

3d. Of *Water* as a cause of epidemics. Water used in drinks is often the cause of endemical diseases, as Hippocrates has shewn with unrivalled ability;† but it is not often in this way that it operates in producing epidemics, but rather by giving rise to noxious vapours or miasmata. Dr. MEAD says, "The waters, especially of lakes and morasses, frequently exhale pestilential vapours, which infect the circumambient air." From this cause, by the admission of our best authorities, both ancient and modern, most intermittent and remittent fevers derive their origin: with these we may rank the malaria which is engendered by exhalations from the Pontine and other marshes of Italy. It is nearly allied to the autumnal remittent fever, so excellently described by Drs. PRINGLE and MUNRO, which, as the latter remarks, "is most frequent and most fatal when a country is covered with wood or is marshy, and when there are frequent fogs and much stagnating water, which corrupts the heat of the summer.‡ ERNDL attributes the Polish plague of 1710 in a great measure to the putrid exhalations from marshes and rivers, the vegetable matters in which had become corrupted, owing to the hot and humid constitution of the atmosphere. ASSALINI informs us, that the epidemic fever which attacked the French hospitals in Italy during the years 1799-1800 was referred, by the majority of the physicians, to the rains and fogs.§ From the most credible statements, it would appear that the Bulam fever, so celebrated in the history of the

* For the same reason, I am inclined to reject the hypothesis now pretty extensively entertained, that the cholera of the present day is propagated by animalculæ in the air. How is it to be supposed that these insects could have lived in such a climate as that of Moscow and other parts of Russia, during the severity of a northern winter?

† De Aeribus, Aquis, et Locis.

‡ On Army Diseases.

§ On the Plague, Dysentery, &c.

yellow fever, derived its origin from the rains and heat of the season.

In Roman history, we read frequently of plagues being consequent upon inundations of the Tyber; and, in modern times, LANCISI ascribes the epidemics in Rome to the same cause. According to AVENZOAR, one of the best of the Arabian authors, many epidemical diseases arise from dead and stagnant waters which have become putrid and fetid.

DIOGENES LAERTIUS relates that the Selinantians, being afflicted with a great pestilence arising from the stench and corruption of a river which surrounded the city, the philosopher EMPEDOCLES, by directing them to convey two neighbouring rivers into it, completely relieved them from the disease.*

4th. Of the *Earth* as a cause of epidemics. The belief that certain distempers rise out of the earth to afflict mankind, is at least as ancient as the poet LUCRETIUS, who expresses himself in the following manner with regard to the origin of pestilential diseases:

Atque ea vis morborum, pestilitasque,

Aut extrinsicus, ut nubes nebulæque superne

Per cœlum veniunt, *aut ipsâ sæpe coorta*

*De terrâ surgunt*, ubi putrorem humida nacta est,

Intempestivis pluviisque et solibus icta.

*De Rerum Natura*, lib. vi.

Here the cause of the pestilence is ascribed to putrefaction engendered in the earth by the combination of heat and moisture. This hypothesis accords very well with that of our SYDENHAM, who states that many epidemics cannot be ascribed to any particular qualities of the atmosphere, and "depend not on heat, cold, drought, or moisture, but on some occult and inexplicable alteration in the very bowels of the earth." If under this head we rank the putrid effluvia from the corruption of animal and vegetable matter, this may be looked upon as by far the most common cause of epidemical distempers; and the records of ancient and modern medicine are so rich in statements and examples to prove its influence, that our only difficulty will consist in making a judicious selection from them.

GALEN says that, in the pestilential constitution, it is the respiration which is principally concerned in propagating the disease, the atmosphere which is respired being tainted

* In Vita Empedoclis. It is not to be concealed, however, that Plutarch ascribes the epidemic in question to a different cause, namely, to noxious gales from a mountain in the neighbourhood of the city. (De Curiositate et Contra Coloton.)

with putrid exhalations, derived either from a multitude of dead bodies which had been left unburied, or from marshes and lakes in the summer season.* The philosopher Plutarch makes mention of a plague which was occasioned by the putrefaction of a whale;† and a similar case is related by Forestus.‡ According to Diodorus Siculus, the plague which attacked the Carthagenian army in Sicily was produced by effluvia from marshes and dead bodies during the heat of summer.§ The historian Appian makes mention of a plague engendered by the corruption of dead bodies.|| Livy gives an account of a similar epidemic:¶ the plague of Amida, described by Ammianus Marcellinus, is referred by him, seemingly with good reason, to the multitude of dead bodies which lay unburied in the town.** So also says the poet Ovid,

"Corpora fœda jacent, vitiantur odoribus auræ."

With regard to the Arabian authors, I may mention briefly that Haly Abbas, Rhases, Avicenna, Alsaharavius, and Avenzoar concur in enumerating the putrid effluvia from animal and vegetable matter among the causes of pestilential diseases. Koesler ascribes pestilential distempers to morbific vapours, either of a putrid nature, or of a sulphurico-arsenical nature, and supposes them to be disengaged from the bowels of the earth by the heat of the sun.†† On the subject of putrefaction, we may remark that several pestilences have been ascribed to flocks of locusts, which had been left dead on certain places: such an event is reported to have happened in Africa, A.D. 118; in Gaul, A.D. 864; and in Italy, A.D. 1478.‡‡ Dr. Assalini explains the origin of the plague which attacked the French army in Syria, by supposing it produced by the putrefaction of the bodies of the Turks which were left dead on the taking of Jaffa, and of the carcases of horses and camels. Similar cases are related by Ambrose Paré and Diemerbroeck. Dr. M'Lean maintains that the epidemical fever of Barcelona, in the year 1821, was occasioned by the putrefaction of animal and vegetable matters in the sewers, cloacæ, and canals of the city. Sir John Pringle refers the hospital fever to the animal steams from foul and diseased bodies.

* De Diff. Febr. i. 6. † De Solertia Animal.

‡ Lib. vi. Observat. 9. See also Paræus, de Peste, c. 3; Agricola, de Peste, lib. i.

§ Biblioth. lib. xiv. || De Bello Punico, lib. vi.

¶ Hist. lib. iii. ** Lib. xix.

†† V. Mangeti Bibl. Scrip. Med., lib. x.

‡‡ For the authorities upon this subject, see Diemerbroeck, de Peste, c. 6; and Prælectiones Marciæ, lib. i.

After all this evidence, we cannot help feeling surprise that the learned Dr. Winterbottom should confidently maintain that "putrefaction does not produce epidemic diseases, or even contagion, nor apparently increase their virulence."* The following extract, however, from the late publication of Dr. Christison on Poisons, contains, as I think, an unanswerable refutation of all such objections. "Putrid animal matter, when injected into the veins of healthy animals, proves quickly fatal; and, from the experiments of Gaspard and Magendie, and the more recent researches of MM. Leuret and Hamont, the disease induced seems to resemble closely the typhoid fever of man. Similar effects were observed by Magendie, when dogs were confined over vessels in which animal matter was decaying, so that they were obliged always to breathe the exhalations. These discoveries throw some light on the question regarding the tendency of putrid effluvia to engender fever in man; and, notwithstanding many well-ascertained facts of an opposite import, it shews that, probably in peculiar circumstances, decaying animal matter may excite epidemic fevers: but the two works quoted below are referred to for examples, in my opinion, of the unequivocal origin of continued fever in the cause now alluded to." (P. 476.) He refers to Mem. de la Soc. Roy. de Med. i. 97, and London Med. Chirurg. Rev. vi. 202. I would further refer to the Westminster Review, No. v. p. 151.

Under this head I must not omit to mention that earthquakes, by disengaging noxious airs from the earth, have been supposed to contribute to the formation of pestilential diseases. Fracastorius mentions that earthquakes are frequently the precursors of the plague; and Dr. Russel states that frequent shocks of earthquakes preceded the fatal plague of Aleppo, in 1760. Irebellius Pollio, one of the writers of the Augustan history, in noticing the plague which desolated Rome in the reign of Galienus, mentions that it was accompanied by an earthquake. To conclude this part of our subject, Croanen, in his Commentary on the works of Henricus Regius, delivers it as his opinion that the pestilential constitution of the atmosphere is occasioned by effluvia from the bosom of the earth, marshes, lakes, and pits, being raised by earthquakes or the power of the solar heat, and getting mixed with the blood, either by deglutition or respiration.

(To be continued.) A. 264.

* Edinburgh Medical and Surgical Journal, No. 96.

